# Epidemiological Investigation of Disease Patterns, Accessibility, and Patient Characteristics Following the Introduction of Dermatology Specialty Clinics Within Primary Care Settings in Qatar

**DOI:** 10.7759/cureus.72964

**Published:** 2024-11-04

**Authors:** Muslim A Syed, Sythra Razaq, Ahmed Sameer Alnuaimi

**Affiliations:** 1 Clinical Research Department, Primary Health Care Corporation, Doha, QAT; 2 Clinical Operations Department, Leabaib Health Center, Primary Health Care Corporation, Doha, QAT

**Keywords:** dermatology, family medicine, general practice, primary care, primary health care

## Abstract

Introduction

Dermatological diseases are commonly presented in primary care settings and are associated with health implications and psychiatric comorbidity, which has a negative impact on the overall quality of life of the patients. The service users’ characteristics and the prevalence of the different types of dermatological diseases are an under-researched area in the state of Qatar. The aim of the study is to investigate the prevalence of commonly presenting dermatological diseases such as diseases of the skin and subcutaneous tissues, dermatitis and eczema, and disorders of skin appendages within primary care settings and highlight patient characteristics accessing dermatological healthcare services at primary healthcare centers within its parent organization Primary Health Care Corporation (PHCC).

Methods

The study population included in the research was composed of both Qataris and non-Qataris registered at a PHCC health center between 1 January 2017 and 31 December 2020. The demographic and diagnosis data were extracted from the electronic medical records (EMR) for the defined population. A comparison analysis (reported as odds ratio and inverse odds ratio) was conducted to evaluate the frequency and distribution of cases at "Primary Care Dermatology Patients Care Services" before and after the introduction of dermatology specialty clinics services in PHCC.

Results

A total of 937,553 patients’ data was retrieved for the study registered at a PHCC health center between 1 January 2017 and 31 December 2020. Adults aged 18 to 59 constituted more than two-thirds of the study sample (68.3%, n=640313). The commonly occurring dermatological diseases that were diagnosed during this four-year time period after the introduction of dermatology specialty clinics were namely diseases of the skin and subcutaneous tissue (n=249197, 73%), dermatitis and eczema (n=91653, 27%) and disorders of skin appendages (n=67933, 20%). The probability of having a clinical encounter with neoplasm was increased by 2.17 times after introducing the specialized clinics within PHCC.

Conclusion

The disease pattern of dermatological diseases and patient characteristics present vital health information to better understand the workload and training needs of Family Physicians working in dermatology specialty clinics and to further improve the quality of service and target high-risk patient sub-groups. The findings of the study can also be utilized for future designing and re-designing of the service, particularly in the context of innovative strategies such as complex care management and patients presenting with co-morbidities including dermatological or cutaneous diseases.

## Introduction

The World Health Organization has emphasized over the last five decades the significance of providing comprehensive primary health care and universal health coverage for the populations to achieve desirable health outcomes [1-3]. Evidence suggests that cutaneous or dermatological diseases are commonly presented at primary care, which approximately constitute less than 10% of all these visits [4-7]. Dermatological diseases are associated with health implications and psychiatric comorbidity, which has a negative impact on the overall quality of life of the patients [8-12]. The most common cutaneous diseases presented in primary care clinics mainly include atopic dermatitis, acne vulgaris, cellulitis/abscess, verruca vulgaris, and benign skin lesions [4,13,14]. Despite the significant burden of disease, psychiatric co-morbidities, and cost implications in the context of dermatological disease cases presented within primary care settings, it is often not prioritized as a major public health issue within different healthcare systems [10,12,13].

Moreover, dermatological diseases adversely affect the mental health of patients [11,15,16]. Patients with dermatological diseases often present with psychological issues such as depression, social isolation, low self-esteem, suicidal ideation, and feelings of embarrassment and shame [10-12,15]. Furthermore, the literature suggests that general practitioners providing healthcare services in primary care clinics require additional training as hospital practitioners in dermatology to meet the needs of the patients and improve the quality of the service [17-19]. Hence, it is worthwhile to investigate and explore the prevalence of various dermatological diseases within different primary healthcare systems with due consideration for the socio-demographic characteristics of the service users to better equip and train the family physicians to cater to the healthcare needs of the patients.

Qatar has recently emerged as a leading provider of comprehensive primary healthcare services through its parent organization "Primary Health Care Corporation" (PHCC), which is composed of 31 primary healthcare centers scattered within the country [20]. The registered service users also have provision to dermatological care by introduction of dermatology specialty clinics. To the best of our knowledge, the service users’ characteristics and the prevalence of the different types of dermatological diseases are an under-researched area in the state of Qatar. The aim of the study is to investigate the prevalence and diagnosis of commonly presenting dermatological diseases in primary care settings [4,13,14] and highlight patient characteristics accessing dermatological healthcare services at primary care centers. The objectives of the study also include conducting a comparison analysis to evaluate the frequency and distribution of cases at "Primary Care Dermatology Patients Care Services" before and after the introduction of dermatology specialty clinic services in PHCC. The findings of the study will provide useful information that can be utilized to improve the quality and efficiency of the services, better plan and design future longitudinal studies to investigate health outcomes and patient-reported outcomes associated with dermatological diseases, and further designing and re-designing the services.

## Materials and methods

Context and study settings

Qatar, a peninsular Arab country (with having world’s third-largest natural gas and oil reserves) has recently emerged as a leading primary healthcare provider country that aims to provide comprehensive primary care with due consideration of the fundamental principles outlined within the concept by the World Health Organization. This was mainly achieved by the development of a universal publicly funded primary healthcare service delivered by the PHCC. PHCC is one of the main and most significant primary care providers in the country publicly with 31 health centers. Importantly all the primary health centers are accredited by Accreditation Canada International and distributed across three geographical regions.

Study population and data collection

The study population included in the research was composed of both Qataris (n=286929, 30.6%) and non-Qataris (n=650624, 69.4%) registered at a PHCC health center between 1 January 2017 and 31 December 2020. The demographic and diagnosis data were extracted from the electronic medical records (EMR) for the defined population.

Study variables

The outcome variable is the International Classification of Diseases, Tenth Revision (ICD10) diagnostic categories. The highest two levels of classification were used. The independent (exposure) variables included age, gender, date of clinical encounter, the time duration in relation to the implementation of the specialized dermatology clinic, and the type of consulting physician. There was no specific age range determined as inclusion criteria and all patient data was included who were registered with the service and accessed dermatological services. The exclusion criteria were patients accessing services other than dermatological disease conditions during the specified time period (between 1 January 2017 and 31 December 2020).

Data collection and quality assurance

The PHCC EMR CERNER system (Cerner Corporation, Kansas City, USA) uses SNOMED codes (a systematically organized computer-processable collection of medical terms providing codes, terms, synonyms, and definitions used in clinical documentation and reporting). The PHCC Business Health Intelligence (BHI) department provided the list of variables using custom filters that satisfy the inclusion criteria for the defined study population. The SNOMED codes are mapped to standard ICD10 codes. A total of 2236 unique dermatologic diagnoses were obtained in the current data file. These unique diagnoses and their mapping to ICD10 codes were validated by a senior dermatologist before being categorized into the two successive levels recommended by the ICD10.

Data analysis

Descriptive statistics were used to analyze the population characteristics and were presented as frequency percentage age, gender, type of appointment, and referral practice. A comparison analysis (reported as odds ratio and inverse odds ratio) was conducted to evaluate the frequency and distribution of cases at "Primary Care Dermatology Patients Care Services" before and after the introduction of dermatology specialty clinics services in PHCC. The diagnosis of the cases was reported according to the classification of ICD10 [21]. Multivariate regression models were applied to control for socio-demographic confounding variables (Appendices A-D).

Ethical considerations

The study received ethics approval from the Institutional Review Board of PHCC (reference no.: PHCC/DCR/2020/12/146). The study is a secondary data analysis and presents minimal risk of harm to its subjects. Moreover, to protect patient confidentiality, the data collected were anonymized and none of the subjects’ personal information was available to the research team by which the patients could be identified.

## Results

Demographic details of the study participants

**Figure 1:**
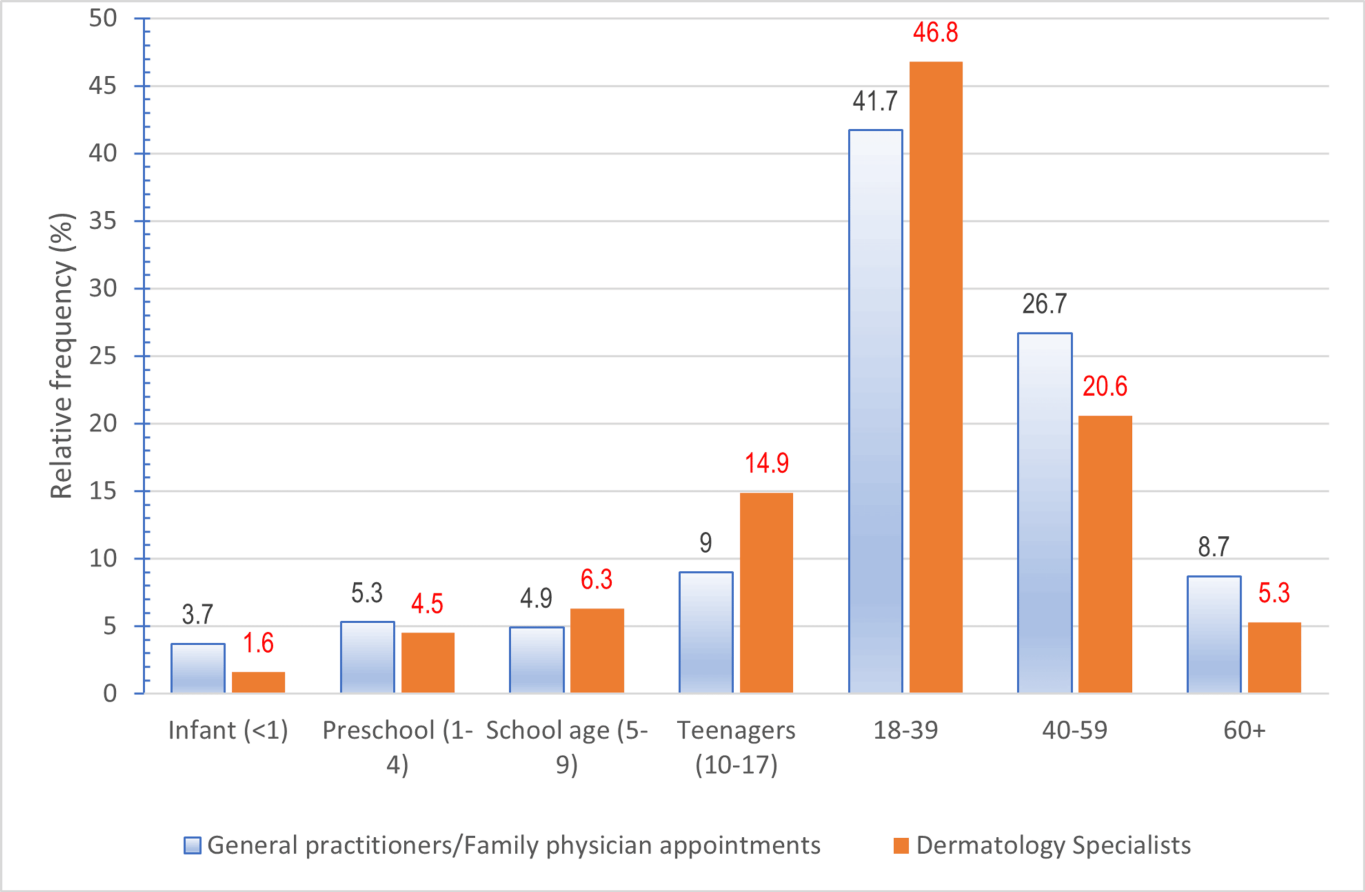
Bar chart comparing the age distribution of clinical encounters between dermatology specialists and other appointments (general practitioners/family physicians).

A total of 937,553 patients’ data was retrieved for the study registered at a PHCC health center between 1 January 2017 and 31 December 2020 (Table 1). Adults aged 18 to 59 constituted more than two-thirds of the study sample (68.3%, n=640313). Female service users accessed service almost twice as frequently (62.2%) compared to males, as depicted in Table 1. The age distribution of clinical encounters between dermatology specialists and other appointments indicated that almost half (46%, n=37215) were between the age groups of 18-39 years of age as illustrated in Figure 1.

**Table 1:** Demographic details of the patients accessing the service between 2017 and 2020 (n=937,553). The males and females add up to 937,523 because there were 30 missing values for information on gender (0.003% which is almost negligible).

Variables	Frequency (n)	Percentage (%)
Age at first diagnosis (years)		
Infant (<1)	33346	3.6
Preschool (1-4)	49102	5.2
School-age (5-9)	46975	5.0
Teenagers (10-17)	88898	9.5
18-39	395058	42.1
40-59	245255	26.2
60+	78919	8.4
Total	937553	100.0
Gender		
Female	583533	62.2
Male	353990	37.8
Total	937523	100.0

Types of appointments and referral practice

During the study period of four years, only 8.6% (n=80903) of clinical encounters of the service users were catered by dermatology specialists as depicted in Table 2. The internal referrals (within the primary healthcare center) to a specialized dermatologist constituted 0.9% (n=8810), while external referrals were only 0.2% (n=1684) of the study sample, as demonstrated in Table 2.

**Table 2:** Frequency distribution of the study sample by type of appointment and referral practice.

Type of appointment and referral practice	Frequency (N)	Percentage (%)
Type of appointment		
General practitioners/family physician appointments	856650	91.4
Dermatology specialists	80903	8.6
Total	937553	100.0
Referral associated with dermatology appointments		
No referral	927059	98.9
Internal-referral	8810	0.9
External-referral	1684	0.2
Total	937553	100.0

Association between launching a specialized dermatology clinic and diagnosis of cutaneous diseases

The probability of having a clinical encounter with neoplasm was increased by 2.17 times after introducing the specialized clinics within PHCC as demonstrated in Table 3. Conversely, the probability of having a clinical encounter with a diagnosis classified under “Chapter IV-Endocrine, Nutritional and Metabolic Diseases” was reduced by 54% (inverse OR=1.54) after introducing the specialized clinics as depicted in Table 3.

**Table 3:** Association between launching a specialized dermatology clinic and the relative frequency of diagnosis of cutaneous diseases (based on ICD10 diagnostic category). Other remaining categories: XIII-Diseases of the musculoskeletal system and connective tissue, Chapter V-Mental and behavioural disorders, Chapter XIV-Diseases of the genitourinary system, Chapter X-Diseases of the respiratory system, Chapter XVIII-Symptoms, signs and abnormal clinical and laboratory findings, not elsewhere classified, Chapter IX-Diseases of the circulatory system, Chapter XVII-Congenital malformations, deformations and chromosomal abnormalities, Chapter III-Diseases of the blood and blood-forming organs and certain disorders involving the immune mechanism. PHCC: Primary Health Care Corporation; OR: odds ratio

	Specialized dermatology clinic launch by PHCC					
International Classification of Disease version 10 (ICD10) diagnostic category	Before		After		OR	Inverse OR
	N	%	N	%		
ICD10 diagnostic categories-Level 1 (top level)	-	-	-	-	-	-
Chapter XII-Diseases of the skin and subcutaneous tissue	409913	69	249197	72.6	1.19	-
Chapter IV-Endocrine, nutritional and metabolic diseases	111558	18.8	45134	13.1	0.65	1.54
Chapter I-Certain infectious and parasitic diseases	71293	12	47465	13.8	1.18	-
Chapter II-Neoplasms	1144	0.2	1435	0.4	2.17	-
Other remaining categories	198	0.03	216	0.06	1.89	-
Total	594106	100	343447	100	-	-
ICD10 diagnostic categories-Level 2	-	-	-	-	-	-
Dermatitis and eczema	175910	29.6	91653	26.7	0.87	1.15
Disorders of thyroid gland	111542	18.8	45125	13.1	0.65	1.54
Disorders of skin appendages	87618	14.7	67933	19.8	1.43	-
Other disorders of the skin and subcutaneous tissue	66294	11.2	44154	12.9	1.17	-
Infections of the skin and subcutaneous tissue	57605	9.7	28717	8.4	0.85	1.18
Mycoses	54910	9.2	27397	8	0.85	1.18
Viral infections characterized by skin and mucous membrane lesions	12646	2.1	17295	5	2.44	-
Urticaria and erythema	16408	2.8	9349	2.7	0.99	1.01
Papulo-squamous disorders	5035	0.8	6427	1.9	2.23	-
Pediculosis, acariasis and other infestations	2932	0.5	1934	0.6	1.14	-
Benign neoplasms	1080	0.2	1356	0.4	2.18	-
Radiation-related disorders of the skin and subcutaneous tissue	929	0.2	894	0.3	1.67	-
Infections with a predominantly sexual mode of transmission	453	0.1	621	0.2	2.37	-
Other bacterial diseases	318	0.1	206	0.1	1.12	-
Other remaining categories	426	0.07	386	0.11	1.57	-
Total	594106	100	343447	100	-	-

Association between type of clinical appointment and the relative frequency of ICD10 diagnostic category

The assessment of the difference in diagnostic categories of clinical encounters (appointments) achieved by the specialized dermatology clinic (SDC) and those by the general practitioners/family physicians is reported in Table 4. The SDC physicians were more than four times more likely to set a diagnosis of neoplasms compared to general practice. Conversely, the diagnoses classified under “Chapter IV-Endocrine, Nutritional and Metabolic Diseases” were almost absent with the SDC physicians, which were more than 1000 times less likely to coin such a diagnostic category compared to general practice (Table 4). The remaining two diagnostic categories were also more frequent with the SDC compared to general practice (diseases of the skin and subcutaneous tissue together with certain infectious and parasitic diseases) as depicted in Table 4.

**Table 4:** Association between type of clinical appointment and the relative frequency of ICD10 diagnostic category. ICD10: International Classification of Disease version 10; OR: odds ratio

	Type of appointment					
ICD diagnostic categories-Level 1 (top level)	General practitioners/family physician appointments		Dermatology specialists		OR	Inverse OR
	N	%	N	%		
Chapter XII-Diseases of the skin and subcutaneous tissue	595230	69.5	63880	79	1.65	-
Chapter IV-Endocrine, nutritional, and metabolic diseases	156675	18.3	17	0	0.001	1000
Chapter I-Certain infectious and parasitic diseases	102587	12	16171	20	1.84	-
Chapter II-Neoplasms	1829	0.2	750	0.9	4.37	-
Other remaining categories	329	0.04	85	0.11	2.74	-
Total	856650	100	80903	100	-	-

## Discussion

Main findings of the study

The key findings of the study mainly include that female service users accessed dermatology specialty clinics almost twice as frequently in comparison with male service users; most cutaneous disease cases were seen in the age groups of 18-39 years of age; four-year assessment of the service revealed that out of the total service users registered at primary health centers within PHCC, only 8.6% (N=80903) visited the dermatology clinics; and the probability of having a clinical encounter with neoplasm was increased by 2.17 times after introducing the specialized clinics within PHCC. The commonly occurring dermatological diseases that were diagnosed during this four-year time period after the introduction of dermatology specialty clinics were namely disorders of the skin and subcutaneous tissue, dermatitis and eczema, and disorders of skin appendages.

Comparison of the findings of the study with existing literature

The study highlighted that female service users registered at primary healthcare centers are more likely to access dermatology specialty clinics. These findings are substantiated by existing literature, which suggests that female patients are more likely to access dermatology clinics within primary care [22]. Interestingly evidence also suggests that cutaneous diseases significantly impact the quality of life of female patients and the associated psychological implications are exacerbated among them [23-25]. The study also documented that patients falling in the age groups of 18-39 were highest in frequency who visited the dermatology clinics. Moreover, studies suggest that dermatology diseases are presented in primary care clinics mostly by patients who fall in younger age groups [26,27]. Although the dermatology visits constituted only 8.6% (n=80903) of the total accessibility of primary health services, it does indicate exploration into the training needs of the family physicians to better manage the patients presenting with cutaneous diseases. This is supported by evidence that highlights that it is important to determine the training requirements of family physicians to diagnose and treat dermatological diseases within primary care settings [17-19].

Furthermore, the study highlighted dermatitis and eczema, disorders of skin appendages, and other disorders of skin and subcutaneous tissue as some of the common cutaneous diseases that were diagnosed in specialty clinics. Similarly, studies indicate that the most common cutaneous diseases presented in primary care clinics mainly include atopic dermatitis, acne vulgaris, cellulitis/abscess, verruca vulgaris, and benign skin lesions [4,13,14].

Recommendations for future research and health policy implications

The following recommendations are suggested for future research based on the findings of the study:

Health Education Campaigns to Increase Accessibility

The study highlighted that only less than 10% of the total visits to primary healthcare centers constituted dermatology specialty clinics. The accessibility to the service can be increased by various health education campaigns to increase awareness about the availability of the service and the health implications associated with dermatological diseases. This can be achieved by face-to-face interactions during clinical consultations, conducting seminars and symposiums within healthcare centers or at-level communities, engaging community members to disseminate the information, initiating capacity-building and training programs aimed at health advocates within the community and using various social media mediums, local newspapers, and health-related television programs to market and promote the service.

Assessment of Training Needs of Family Physicians Running Dermatology Specialty Clinics

It is important to assess the training needs of family physicians operating within dermatology specialty clinics to improve the quality of the service. Such assessments will contribute towards the professional development of family physicians and will lead to more accurate diagnoses and better management of patients presenting with various cutaneous diseases. Routine surveys and audits can be conducted to ensure that these requirements are met. Validated tools can be utilized to assess the competency levels of family physicians and compare them with the desired set of skills required to manage patients with dermatological conditions. The information can feed into the continuous professional development training program (in the broader context of medical education) designed to meet the training needs of family physicians to better manage patients.

Promoting Focused Care for High-Risk Groups

Future research can be focused on high-risk patient groups, particularly those with co-morbidities associated with cutaneous diseases presenting in the dermatology specialty clinics. Such measures can contribute towards complex case management, promote patient-centered care, and increase the overall efficiency of the service.

Investigating the Health Literacy Levels of the Service Users Registered With Primary Healthcare Corporation

It will also be worthwhile to conduct further research to capture preferences and perceptions regarding the health literacy of patients in the context of their general well-being (physical and mental health) and the management of dermatological disease conditions. Research can also be conducted to highlight the various mediums of health education that are currently being utilized and explore the gaps in practice from a patient’s perspective.

Designing Longitudinal and Interventional Study Designs to Capture Disease Outcomes and Patient-Reported Outcomes

The present study was a cross-sectional study that aimed to analyze health service delivery for dermatology-related diagnoses. Future longitudinal studies and interventional designs can be conducted to capture dermatological disease outcomes and capture patient-reported outcomes utilizing generic and dermatological disease-specific patient-reported outcome measures (PROMs) such as Dermatology Life Quality Index (DLQI) and Skindex-16 to capture the quality of life of patients with dermatological diseases [28]. Such studies can be designed within the primary healthcare settings of Qatar and similar healthcare settings within the eastern Mediterranean region and countries with high expatriate populations. As previously discussed, dermatological diseases are associated with mental health issues and this can be further explored by conducting an in-depth qualitative study to explore the perceptions of patients and the socio-cultural aspects that might influence the psychological and psychiatric comorbidity associated with dermatological diseases.

Strengths and limitations of the study

One of the key strengths of the study is that the inferences drawn from the study are based on patient data of nearly a million registered patients at PHCC spanning over a period of four years. This is significant data and strengthens the generalizability of the findings of the study. However, the data is retrieved from 2017 to 2020 and it can argued, that the findings might slightly change if the study were to be conducted at present. However, the findings of the study are substantiated by studies conducted in the last three years and the recommendations and health policy implications are applicable for future research.

One of the main limitations of the study is that the study applied a cross-sectional study design and aimed to analyze health service delivery for dermatology-related diagnoses. The outcome of treatment was not addressed. A longitudinal design could have been applied to capture outcomes. However, the study provides a basis for future studies, which can be designed primarily focusing on patient outcomes as well as applying generic and disease-specific PROMs to capture patient satisfaction and mental health concerns regarding dermatological diseases.

## Conclusions

The disease pattern of dermatological diseases and patient characteristics present vital health information to better understand the workload and training needs of family physicians working in dermatology specialty clinics and to further improve the quality of service and target high-risk patient sub-groups. The findings of the study can also be utilized for future designing and re-designing of the service, particularly in the context of innovative strategies such as complex care management and patients presenting with co-morbidities including dermatological or cutaneous diseases.

## Appendices

Appendix A

**Table 5:** Multiple logistic model with the odds of having a diagnosis classified as “Chapter XII-Diseases of the skin and subcutaneous tissue” as the outcome variable by age, gender and changes introduced after launching the SDC. P (Model) < 0.001; ** not applicable Predictive accuracy = 70.3% SDC: specialized dermatology clinic; OR: odds ratio; CI: confidence interval

Exploratory Variables	Partial (adjusted) OR	95% CI OR	P
Being 18-39 years compared to those younger than 18 years	0.45	(0.45 to 0.46)	<0.001
Being 40-59 years compared to those younger than 18 years	0.31	(0.3 to 0.31)	<0.001
Being 60+ years compared to those younger than 18 years	0.3	(0.29 to 0.31)	<0.001
Males compared to females	1.58	(1.57 to 1.6)	<0.001
Changes introduced after launching specialized dermatology clinics compared to the period before that	1.12	(1.11 to 1.14)	<0.001
Constant	4.25	**	<0.001

Appendix B

**Table 6:** Multiple logistic model with the odds of having a diagnosis classified as “Chapter IV-Endocrine, nutritional and metabolic diseases” as the outcome variable by age, gender and changes introduced after launching the SDC. P (Model) < 0.001; ** not applicable Predictive accuracy = 83.3% SDC: specialized dermatology clinic; OR: odds ratio; CI: confidence interval

Exploratory Variables	Partial (adjusted) OR	95% CI OR	P
Being 18-39 years compared to those younger than 18 years	9.14	(8.86 to 9.42)	<0.001
Being 40-59 years compared to those younger than 18 years	14.97	(14.52 to 15.44)	<0.001
Being 60+ years compared to those younger than 18 years	14.78	(14.28 to 15.29)	<0.001
Males compared to females	0.26	(0.26 to 0.26)	<0.001
Changes introduced after launching specialized dermatology clinics compared to the period before that	0.69	(0.68 to 0.69)	<0.001
Constant	0.038	**	<0.001

Appendix C 

**Table 7:** Multiple logistic model with the odds of having a diagnosis classified as “Chapter I-Certain infectious and parasitic diseases” as the outcome variable by age, gender and changes introduced after launching the SDC. P (Model) < 0.001; ** not applicable Predictive accuracy = 87.3% SDC: specialized dermatology clinic; OR: odds ratio; CI: confidence interval

Exploratory ariables	Partial (adjusted) OR	95% CI OR	P
Being 18-39 years compared to those younger than 18 years	0.95	(0.93 to 0.96)	<0.001
Being 40-59 years compared to those younger than 18 years	1.03	(1.01 to 1.05)	0.002
Being 60+ years compared to those younger than 18 years	1.13	(1.11 to 1.16)	<0.001
Males compared to females	1.65	(1.63 to 1.67)	<0.001
Changes introduced after launching specialized dermatology clinics compared to the period before that	1.19	(1.17 to 1.2)	<0.001
Constant	0.11	**	<0.001

Appendix D 

**Table 8:** Multiple logistic model with the odds of having a diagnosis classified as “Chapter II-Neoplasms” as the outcome variable by age, gender and changes introduced after launching the SDC. P (Model) < 0.001; ** not applicable Predictive accuracy = 99.7% SDC: specialized dermatology clinic; OR: odds ratio; CI: confidence interval

Exploratory variables	Partial (adjusted) OR	95% CI OR	P
Being 18-39 years compared to those younger than 18 years	1.52	(1.37 to 1.7)	<0.001
Being 40-59 years compared to those younger than 18 years	1.75	(1.56 to 1.96)	<0.001
Being 60+ years compared to those younger than 18 years	1.27	(1.07 to 1.5)	0.007
Males compared to females	1.22	(1.13 to 1.32)	<0.001
Changes introduced after launching specialized dermatology clinics compared to the period before that	2.23	(2.06 to 2.41)	<0.001
Constant	0.001	**	<0.001

## References

[1] Abbas SM, Alam AY, Malik MR (2012). Primary health care: what is it and what is it not? Views of teaching faculty at an undergraduate medical college in Pakistan. East Mediterr Health J.

[2] Muldoon LK, Hogg WE, Levitt M (2006). Primary care (PC) and primary health care (PHC). What is the difference?. Can J Public Health.

[3] Cueto M (2004). The origins of primary health care and selective primary health care. Am J Public Health.

[4] Seth D, Cheldize K, Brown D, Freeman EF (2017). Global burden of skin disease: inequities and innovations. Curr Dermatol Rep.

[5] Ferreira IG, Godoi DF, Perugini ER (2020). Nosological profile of dermatological diseases in primary health care and dermatology secondary care in Florianópolis (2016-2017). An Bras Dermatol.

[6] Symvoulakis EK, Krasagakis K, Komninos ID, Kastrinakis I, Lyronis I, Philalithis A, Tosca AD (2006). Primary care and pattern of skin diseases in a Mediterranean island. BMC Fam Pract.

[7] Salava A, Oker-Blom A, Remitz A (2021). The spectrum of skin-related conditions in primary care during 2015-2019-a Finnish nationwide database study. Skin Health Dis.

[8] Oral H (2014). A seat at the big table: expanding the role of dermatology at the World Health Organization and beyond. J Invest Dermatol.

[9] Hay RJ, Johns NE, Williams HC (2014). The global burden of skin disease in 2010: an analysis of the prevalence and impact of skin conditions. J Invest Dermatol.

[10] Harlow D, Poyner T, Finlay AY, Dykes PJ (2000). Impaired quality of life of adults with skin disease in primary care. Br J Dermatol.

[11] Picardi A, Mazzotti E, Pasquini P (2006). Prevalence and correlates of suicidal ideation among patients with skin disease. J Am Acad Dermatol.

[12] Gupta MA, Gupta AK (2003). Psychiatric and psychological co-morbidity in patients with dermatologic disorders: epidemiology and management. Am J Clin Dermatol.

[13] Castillo-Arenas E, Garrido V, Serrano-Ortega S (2014). Skin conditions in primary care: an analysis of referral demand. Actas Dermosifiliogr.

[14] Wilmer EN, Gustafson CJ, Ahn CS, Davis SA, Feldman SR, Huang WW (2014). Most common dermatologic conditions encountered by dermatologists and nondermatologists. Cutis.

[15] Cortés H, Rojas-Márquez M, Del Prado-Audelo ML, Reyes-Hernández OD, González-Del Carmen M, Leyva-Gómez G (2022). Alterations in mental health and quality of life in patients with skin disorders: a narrative review. Int J Dermatol.

[16] Duman H, Topal IO, Kocaturk E (2016). Evaluation of anxiety, depression, and quality of life in patients with acne vulgaris, and quality of life in their families. Dermatologica Sinica.

[17] El-Azim AA, El-Wahed MA, Ishish SM (2014). Pattern of dermatologic care by family physicians versus dermatologists. Menoufia Med J.

[18] Clark RA, Rietschel RL (1983). The cost of initiating appropriate therapy for skin diseases: a comparison of dermatologists and family physicians. J Am Acad Dermatol.

[19] Gerbert B, Maurer T, Berger T (1996). Primary care physicians as gatekeepers in managed care. Primary care physicians' and dermatologists' skills at secondary prevention of skin cancer. Arch Dermatol.

[20] El Zoghbi M, Farooq S, Abulaban A (2021). Improvement of the patient safety culture in the primary health care corporation-Qatar. J Patient Saf.

[21] (2024). National Center for Health Statistics ICD-10. https://www.cdc.gov/nchs/icd/icd-10/index.html#cdc_generic_section_5-accessing-icd-10.

[22] Hai J, Nguyen M, Kim-Lim P, Wa Cheung K, Jan R, Tartar DM (2021). Characteristics of patients seen at a dermatology free clinic, 2017-2020: a retrospective chart review. Dermatol Online J.

[23] Schmitt JV, Ribeiro CF, Souza FH, Siqueira EB, Bebber FR (2012). Hair loss perception and symptoms of depression in female outpatients attending a general dermatology clinic. An Bras Dermatol.

[24] Sutton AV, Ellis CN, Spragg S (2017). Improving patient satisfaction in dermatology: a prospective study of an urban dermatology clinic. Cutis.

[25] Julian CG (1999). Dermatology in general practice. Br J Dermatol.

[26] De Vere Hunt I, Chapman K, Wali G, Bullus S, Fisher R, Matin RN, McPherson T (2019). Establishing and developing a teenage and young adult dermatology clinic with embedded specialist psychological support. Clin Exp Dermatol.

[27] Cohen AD, Dreiher J, Vardy DA, Weitzman D (2008). Nonattendance in a dermatology clinic - a large sample analysis. J Eur Acad Dermatol Venereol.

[28] Szabó Á, Brodszky V, Rencz F (2022). A comparative study on the measurement properties of Dermatology Life Quality Index (DLQI), DLQI-Relevant and Skindex-16. Br J Dermatol.

